# Antiamnesic and Neurotrophic Effects of *Parkia biglobosa* (Jacq.) R. Br (Fabaceae) Aqueous Extract on In Vivo and In Vitro Models of Excitotoxicity

**DOI:** 10.1155/bn/8815830

**Published:** 2025-01-06

**Authors:** Antoine Kavaye Kandeda, Liliane Yimta Foutse, Corneille Tongoue, Jean Philippe Djientcheu, Théophile Dimo

**Affiliations:** ^1^Department of Animal Biology and Physiology, University of Yaoundé I, Yaoundé, Cameroon; ^2^Department of Pharmacy, University of Montagnes, Bangangté, Cameroon

**Keywords:** amnesia, neurogenesis, *Parkia biglobosa*, scopolamine

## Abstract

Amnesia is a memory disorder marked by the inability to recall or acquire information. Hence, drugs that also target the neurogenesis process constitute a hope to discover a cure against memory disorders. This study is aimed at evaluating the antiamnesic and neurotrophic effects of the aqueous extract of *Parkia biglobosa* (*P. biglobosa*) on in vivo and in vitro models of excitotoxicity. For the in vivo study, 42 adult male rats were divided into six groups of seven rats each and treated daily for 30 days as follows: normal control group (distilled water, 10 mL/kg, po), negative control group (distilled water, 10 mL/kg, po), positive control group (piracetam, 200 mg/kg, po), and 03 test groups (extract, 44, 88, and 176 mg/kg, po). Scopolamine (0.5 mg/kg, ip) was administered once daily, 45 min after these treatments, for 14 days, except in the normal control group. The animals were then subjected to short-term memory (new object recognition and T-maze) and long-term memory (radial arm maze) tests for 15 following days. Animals were then euthanized, and biochemical analyses (neurotransmitters, oxidative status, and neuroinflammation) were performed in the prefrontal cortex, hippocampus, and serum. Histological analysis of these organs was also carried out. In the in vitro study, the effect of the extract (5, 10, 19, 40, 77, 153, 306, 615, 1225, and 2450 *μ*g/mL) was assessed on the viability of primary cortical neurons exposed to L-glutamate (0.1 mg/mL). Scopolamine induced memory impairment and increased oxidative stress, neuroinflammation, and neuronal loss. *P. biglobosa* extract (44 mg/kg) reduced (*p* < 0.001) short- and long-term memory deficit. It also increased (*p* < 0.01) the concentration of acetylcholine, reduced (*p* < 0.001) that of malondialdehyde, and limited (*p* < 0.001) neuroinflammation and neuronal loss (*p* < 0.001). In addition, the extract (2450 *μ*g/mL) increased (*p* < 0.001) the percentage of viable cells. These results suggest that the extract has effects on amnesia and neurogenesis. These effects seem to be mediated by antioxidant and anti-inflammatory modulations.

## 1. Introduction

Amnesia is a memory disorder in which the subject is unable to recall or acquire information [1]. Amnesia is characterized by a transient memory deficit, which may be followed by progressive loss of other cognitive functions, including behavioral changes [1]. Parkinson's disease and Alzheimer's disease (AD) represent the most common types of dementia in adults [2]. The worldwide prevalence of dementia in people aged over 60 is estimated at around 3.9% [3]. Regional prevalence is 6.4% in North America, 4.6% in Latin America, 3.9% in Eastern Europe, 5.4% in Western Europe, 4.0% in China, and 1.6% in Africa [3]. According to the United Nations, the number of age-related cases could rise from 25.5 million in 2000 to around 114 million in 2050 [3]. Memory disorders are multifactorial and may result from head trauma, metabolic disorders, seizures, psychological disorders, or neurodegenerative diseases [1]. Memory disorders particularly observed in AD have been characterized by the loss of cholinergic neurons and low levels of the neurotransmitter acetylcholine (ACh). Acetylcholinesterase (AChE) and butyrylcholinesterase (BChE) cause the degradation of ACh, which is responsible for memory challenges. Nowadays, drug treatments are not very effective in treating memory disorders and have many undesirable side effects. Phytotherapy has proved to be an increasingly popular treatment option for this condition, thanks to its wealth of active molecules and minor side effects [4]. In Africa, particularly in Cameroon, many plants have shown potential antiamnesic properties, such as *Drymaria cordata* [4, 5].

*Parkia biglobosa* (*P. biglobosa*), a plant for medicinal use, is our plant of interest. It is a leguminous member of the Fabaceae family, native to West and Central Africa [6]. Medicinally, it is used to treat amoebiasis, hookworm, asthma, sterility, malaria, cardiac disorders, gastric ulcers, and dental pain [7]. It is also used to treat neuralgia and memory disorders in the northern region of Cameroon [7]. Studies have documented its nootropic, anxiolytic, and neuroprotective effects [7]. This diversity in treatment could be due to the presence of numerous secondary metabolites such as alkaloids, anthraquinones, flavonoids, saponins, and triterpenoids [6, 7]. The work of Kandeda et al. [7] demonstrated the plant's antiamnesic and anxiolytic effects in a pentylenetetrazole (PTZ) induction model. Ethnobotanical surveys of *P. biglobosa* in Africa have revealed antioxidant activity and an antidepressant effect [8]. Based on this potential of the extract, we hypothesized that the aqueous extract of *P. biglobosa* can mitigate memory impairment and stimulate the neurogenesis process. Thus, this study is aimed at evaluating the antiamnesic and neurotrophic effects of the aqueous extract of *P. biglobosa* using in vivo and in vitro models of excitotoxicity. More specifically, it was to evaluate the antiamnesic effects of *P. biglobosa* aqueous extract using scopolamine (SCO)-induced memory impairment in rats; to determine the effects of *P. biglobosa* aqueous extract on some biochemical markers of memory disorders (ACh, AChE, and gamma amino butyric acid (GABA)), oxidative stress (malondialdehyde (MDA) and reduced glutathione (GSH)), and neuroinflammation (tumor necrosis factor-alpha) in the prefrontal cortex, hippocampus, and serum; to assess the effects of *P. biglobosa* extract on SCO-induced injuries in the hippocampus and prefrontal cortex; and finally, to evaluate in vitro the neurotrophic effects of *P. biglobosa* aqueous extract on primary neurons.

## 2. Materials and Methods

### 2.1. Plant Collection, Extraction, and Doses

The bark of *P. biglobosa* (Fabaceae) was collected in Ngaoundéré (Adamaoua Region of Cameroon). Botanical identification was carried out by Mr. Christian Yombo at the National Herbarium of Cameroon in comparison with a sample kept under Reference Number N°58968/HNC. Preparation of the extract was carried out according to the procedure indicated by a traditional healer. Briefly, *P. biglobosa* bark was harvested, cleaned, shade-dried, and ground to a fine powder. One hundred and forty-four (144) grams of *P. biglobosa* powder was added to a container containing 6 L of distilled water, then boiled for 30 min. After cooling to room temperature, the resulting mixture was filtered through Whatman No. 1 filter paper and dried at 45°C in an oven. This operation yielded 21.73 g of dry extract, equivalent to a yield of 15.09%. The mass obtained enabled us to calculate the human equivalent dose (HED) of 14.28 mg/kg according to the following relation:

 $$\begin{array}{c} \text{ HED}=\frac{\text{mass of dried extract }\left(\text{mg}\right)}{70\;\left(\text{kg}\right)} \end{array}$$

The extract doses to be administered to rats were determined using the interspecies dose interpolation formula. This is expressed by multiplying the HED by a conversion factor (6.17):

 $$\begin{array}{c} \text{animal dose }\left(\text{AD}\right)=\text{HED}\times 6.17 \end{array}$$

Using this formula, we obtained an AD of 88 mg/kg, which was then framed by two doses of 44 and 176 mg/kg. These doses were obtained, respectively, by dividing the AD by 2 and multiplying the AD by 2.

### 2.2. Drugs and Chemicals

The SCO used is manufactured by Thermo Scientific (Bremen, Germany). The piracetam used as a reference substance has the trade name Nootropyl and is manufactured by Union Chimique Belge (UCB Pharma, Bruxelles, Belgium). We also used 0.9% sodium chloride solution and Tris hydrochloride (HCl) buffer (Sigma–Aldrich, St Louis, United States).

### 2.3. Animals and Housing Conditions

The animals used in this study were adult male Wistar rats aged 9–11 weeks.

Male Wistar rats were chosen because sex differences can influence the results of neurobiological studies. Females have a fluctuating menstrual cycle (fluctuating hormones), making data difficult to interpret and results more variable. In addition, female hormones have both a memory-enhancing effect and an antiamnesic effect, making it very difficult to distinguish the effect of a test product from that of the hormone. These animals were bred at the Animal Physiology Laboratory of the University of Yaoundé I and kept in cages with five animals per cage, under a natural cycle (12 h of light and 12 h of darkness). They had free access to food and water. For 50-kg bags, their food consisted of 1% multivitamin complex, 4% bone powder, 5% peanuts, 10% wheat, 20% smoked fish, and 60% corn. A pregnant Wistar rat was also used for in vitro manipulation. This animal had a litter of 8–18-day-old rat embryos. All studies were carried out following national (No. FWA-IRB00001954) ethics governing the use of laboratory animals. All animal experiments comply with the Animal Research: Reporting of In Vivo Experiments (ARRIVE) guidelines.

### 2.4. Experimental Design and Treatment

In this study, the animals were divided into six groups of seven rats each and treated as follows (Table 1):

i. A normal control group given distilled water (10 mL/kg, po),
ii. A negative control group given distilled water (10 mL/kg, po),
iii. A positive control group which received piracetam (200 mg/kg, po), and
iv. 03 test groups receiving the aqueous extract of *P. biglobosa* (44, 88, and 176 mg/kg, po).

The treatments were administered as a single daily dose for 30 days. Intraperitoneal administration of SCO (0.5 mg/kg, ip) was performed daily for the first 15 days, 45 min after administration of the different solutions, except for the normal control group (distilled water 10 mL/kg, ip).

**Figure 1:**
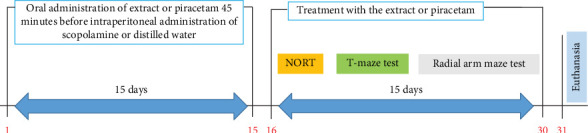
Schematic diagram of the in vivo experimental procedure. NORT: novel object recognition test.

The animals were then subjected to behavioral tests of short-term memory (new object recognition and T-maze tests) and long-term memory (radial arm maze test) for 15 other days. Behavioral test data were recorded using the ANY-Maze 7.0 application. Following the behavioral tests, the animals were euthanized, and their brains were removed for biochemical (*n* = 5) and histological (*n* = 2) analyses (Figure 1).

### 2.5. Behavioral Studies

#### 2.5.1. Novel Object Recognition Test

The open arena is a square enclosure with high edges, illuminated at its center, which prevents the animal from either escaping or hiding. The device used (40 cm long × 40 cm wide × 45 cm high) is similar to that described by Penda et al. [9]. The exploration surface of this enclosure is divided into 17 squares of equal size (10 × 10 cm): 16 squares dividing the inner surface of the device and one central square.

The test of recognition of a new object comprises three phases: habituation, acquisition, and retention. It took place over 3 days:

- The first day of the test was dedicated to habituation. Rats were individually introduced into the open arena for 5 min to reduce stress due to neophobia;
- The second phase of the test, or acquisition phase, began 24 h after the habituation session and lasted 5 min for each rat. The animals were placed in the presence of two identical objects (*A* + *A*) located at opposite corners of the open arena for free exploration;
- The retention or test phase took place 24 h after the acquisition phase. This phase was carried out in the same way as the acquisition phase, except that an old object (*A*) was replaced by a new object (*B*). The animal is considered to be exploring when it moves towards the object (A or B). The exploration times of the two objects (*A* and *B*) were recorded and noted tA and tB, respectively, for 5 min. The recognition index (RI) was therefore calculated according to the following formula:

 $$\begin{array}{c} \text{RI}=\frac{\text{tB}}{\text{tA}+\text{tB}}\times 100 \end{array}$$

where RI stands for recognition index, tA stands for time to explore object *A*, and tB stands for time to explore object *B*.

#### 2.5.2. T-Maze Test

The device used in this study is a T-shaped three-branch maze, consisting of a start compartment, a central aisle, and two finish compartments, both opposite each other. The start compartment (30 cm long × 10 cm wide × 20 cm high) and the finish compartment (30 cm long × 10 cm wide × 20 cm high) are separated from the central aisle by manually operated sliding doors. The test ran for 3 days. The first day was dedicated to the habituation phase. Prior to this, the animals underwent 24 h of food restriction to keep them at 80%–85% of their body weight. Each animal was familiarized with the device for a period of 5 min. For this, a food reinforcer was placed in each of the arrival arms of the maze to encourage exploration. Each rat was individually placed in the starting compartment. After 15 s, the sliding doors giving access to the maze arrival arms were opened, allowing the animal to enter any of the maze arrival arms to determine its preferred arm. The following parameters were recorded for each rat:

- The latency time to choose an arm,
- The arrival arm chosen in first,
- The number of entries in the arrival arms,
- The time spent in each arm.

The second day was dedicated to the acquisition phase. The lane of the arm discriminated by the animal was closed, and a reinforcer was placed in the lane of the arm chosen by the rat. Each animal was placed in the starting arm for 5 min of free exploration.

The third day was dedicated to the retention phase. For a duration of 5 min, each rat was placed in the starting arm and all arms of the device were opened. Food was placed in both arrival arms, and the following parameters were recorded for each rat:

- The number of entries into the preferred and discriminated arms,
- The time spent in the preferred and discriminated arms.

#### 2.5.3. Radial Arm Maze Test

Before the start of the experiment, the animals underwent mild food restriction (7-h fasting) to induce appetite [10]. The protocol comprised two phases: a familiarization phase and a retention phase.

The familiarization phase took place over 3 days. On the first day of the familiarization phase, rats were introduced in groups of three to the maze for 15 min in the presence of food dispersed throughout the platform. On the second day, food was restricted to Arms 1 and 3 of the maze, and animals were placed individually in the maze for 5 min. On the final day of familiarization, each animal was placed individually for 5 min and a bait was placed only in Arms 2, 4, 6, and 8.

The retention phase took place over 5 days, similar to the last day of familiarization, and the following parameters were recorded: the time taken by the animal to visit the baited and unbaited arms. If, after 5 min, the animal had not managed to visit all the baited arms, it was removed from the maze and this maximum time was considered the animal's data. Working memory error and reference memory error were then determined. The rat's entry into arms that had never been baited was marked as a reference memory error, and entry into arms where the food reward would have been eaten was recorded as a working memory error.

### 2.6. Biochemical Analysis

#### 2.6.1. Animal Euthanasia and Preparation of Homogenates

At the end of the treatment period and behavioral tests, the animals were euthanized by decapitation, and the blood was collected in dry tubes. The brains of each animal were removed, washed in 0.9% NaCl, and wrung out on toilet paper. Brains were divided into three per batch. The hippocampi and prefrontal cortices of part of the brains were isolated after hardening on ice for 15 min. These organs were weighed and placed in a ceramic mortar into which Tris HCl 50 buffer (mM; pH = 7.4) was added to prepare 20% homogenates. Dry tubes containing blood were centrifuged at 3000 rpm at 4°C for 15 min and homogenates at 3000 rpm at 4°C for 25 min. Supernatants were, respectively, collected and stored at −20°C for evaluation of biochemical parameters. Some of the brains were fixed in 4% formalin for histological sectioning.

#### 2.6.2. Concentration of ACh

ACh content was estimated by the Hestrin method as described by Cadwell et al. [11]. Homogenate 0.8 mL was taken and placed in dry tubes. Subsequently, 1.4-mL distilled water, 0.2 mL physostigmine (1.5 mM), and 0.8 mL trichloroacetic acid (TCA) (20%) were added. The resulting mixture was centrifuged, and 1 mL of supernatant was collected. After this step, 1 mL of basic hydroxylamine was added to dry tubes, followed by 1 mL of the supernatant. The resulting mixture was incubated for 15 min at 25°C. After incubation, 0.5 mL HCl (4 M) and 0.5 mL ferric chloride (FeCl_3_) (0.37 M) were added to the mixture. Absorbance was read at 540 nm against the blank with a spectrophotometer, and ACh content was expressed in micromole per gram tissue weight.

#### 2.6.3. Activity of AChE

AChE activity was determined by Ellman's method [12]. Twenty microliters of Tris HCl buffer (0.1 M, pH 8.0) and 2 mL of Ellman's reagent were introduced in the tubes (test and blank). In the test tubes, 100 *μ*L of homogenate was added and 100 *μ*L of Tris HCl buffer (HCl 50 mM; potassium chloride (KCl) 150 mM; pH 7.4) in the blank tubes. The tubes were topped up with 20 *μ*L of acetylthiocholine iodide (30 mM). The resulting mixture was homogenized (gentle manual agitation), and a stopwatch was started. Absorbance was read at 412 nm against blank between 30 and 90 s. Enzymatic activity was expressed in micromoles of hydrolyzed acetylthiocholine iodide per gram of tissue per minute.

#### 2.6.4. Concentration of GABA

The amount of GABA in the homogenate was assessed using the colorimetric assay technique described by Chauhan et al. [13]. Briefly, 0.2 mL of ninhydrin solution (0.14 M) was introduced into dry tubes. Subsequently, 0.1 mL of 10% glacial TCA was added to the tubes. One hundred microliters of the homogenate supernatant was then taken and added to the tubes. The resulting mixture was incubated at 60°C in a water bath for 30 mins. After cooling, 2 mL of copper tartrate solution, prepared from 0.16% disodium carbonate, 0.03% copper sulfate, and 0.031% tartaric acid, was added to the mixture. The mixture was then incubated again for 10 min at 25°C. At the end of incubation, 2 mL of glacial TCA (10%) was added to each tube. Absorbance was read at 492 nm using a spectrophotometer. To determine the concentration of GABA in the samples, a calibration curve was generated from increasing concentrations of standard GABA (100, 150, 200, 250, 300, 350, and 400 *μ*mol/g). These solutions were prepared in the same way as above, except that the samples were replaced by standard GABA. The GABA concentration was expressed in micromole per gram.

#### 2.6.5. Concentration of Reduced GSH

Reduced GSH assay was performed according to the protocol described by Fogwe, Reddy, and Mesfin [14]. Two milliliters of Ellman's reagent was introduced into tubes containing 200 *μ*L of homogenates (test tubes) and 200 *μ*L of Tris HCl buffer (0.1 M, pH 7.4) (white tube). The mixture was then homogenized with a shaker and incubated at 37°C for 60 min. Absorbances were read at 412 nm against the blank using a spectrophotometer. The concentration of GSH was expressed as micromole per gram tissue.

#### 2.6.6. Concentration of MDA

MDA content will be measured according to the protocol described by Hannula and Duff [15]. For this analysis, 250 *μ*L of homogenate was introduced into the test tubes and 250 *μ*L of Tris buffer (HCl 50 mM; KCl 150 mM; pH 7.4) into the blank tube. Subsequently, 250 *μ*L of TCA (20%) and 500 *μ*L of TBA (0.67%) were added to all tubes (test and blank). Tubes were capped with glass beads and incubated at 70°C in a water bath for 10 min. After air cooling, tubes were centrifuged at 3000 rpm for 15 min at room temperature. The supernatant was pipetted, and the absorbance was read at 530 nm against the blank with a spectrophotometer. MDA concentration was expressed in micromole per gram protein.

#### 2.6.7. Determination of Proinflammatory Cytokines

As part of this manipulation, specific primary antibodies (10 mg) for each protein were adsorbed to the bottom of dry tubes. The tubes were then incubated at 4°C for 24 h. Each tube was washed five times with wash buffer, and then, 50 *μ*L of RD1-42 dilution solutions for TNF-*α* were introduced into all tubes. Subsequently, 50 *μ*L of Tris buffer (HCl 50 mM; KCl 150 mM; pH 7.4) was added to control tubes and 50 *μ*L of homogenate to test tubes. Tubes were incubated at 37°C for 2 h. After incubation, tubes were again washed five times with wash buffer. One hundred microliters of biotin-conjugated TNF-*α*-specific capture antibody was added. Tubes were incubated a second time at 37°C for 2 h. Again, each tube was washed five times with wash buffer and 100 *μ*L of peroxidase-coupled streptavidin substrate. The tubes were then incubated for 30 min at room temperature, protected from light. The enzymatic reaction was stopped by adding 1.96 mL of stop solution (sulfuric acid (H_2_SO_4_), 0.5 M). Absorbance was read at 405 nm against the blank using a spectrophotometer. TNF-*α* concentration was expressed in picograms per milligram of protein. A calibration curve was made using standard TNF-*α* concentrations.

### 2.7. Histopathological Analysis of Brain Tissues

The histological analysis included fixing, cutting, dehydration, inclusion, cutting, coloring, mounting, and observation. The stained and mounted slides were observed at 250× magnification using a Scientico STM-50 optical microscope (HSIDC Industrial Estate, Haryana, India) equipped with a Celestron 44421 digital camera connected to a computer. Furthermore, the density of neurons in the Cornu Ammonis (CA) 1 and CA3 layers of the hippocampus was determined by counting the number of neurons per 400 *μ*m^2^.

### 2.8. Culture of Primary Cortical Neurons

The method described by Schultz and Engelhardt [16] was used on an 18-day-old pregnant Wistar rat. The animal was sacrificed by cervical dislocation, the rat embryos were extracted, and the cerebral cortex was rapidly harvested after skull incision. The cortexes were minced with a sterile razor blade and diluted in phosphate-buffered saline (0.1 M, pH 7.4) for 15 min. A Pasteur pipette was used for cell dissociation (approx. five to 10 times). After centrifugation (200 rpm for 3 min at 37°C), cells were resuspended in Dulbecco's modified Eagle's medium. Fetal bovine serum 0.9 mL each (15%), glutamine (2 mM), sodium bicarbonate (4.2 mM), bovine serum albumin (0.3 g/L), *β*-mercaptoethanol (0.1 mM), penicillin (1%), and gentamycin (50 pg/mL) were added to this culture medium (petri dish). Cells were cultured on 0.1% poly-L-lysine plates. Culture media were incubated at 37°C in a humidified atmosphere of 5% carbon dioxide (CO_2_). To prevent the proliferation of nonneuronal cells, cytosine HCl *β*-D-arabinofuranoside (10 *μ*M) was added to the medium 3 days after seeding. In this experiment, we used cells after 3 days in culture to assess the neurotrophic activity of the extract in the cell viability assay.

#### 2.8.1. 3-[4,5-Dimethylthiazol-2-yl]-2,5 Diphenyl Tetrazolium Bromide (MTT) Cell Viability Assay

Twenty microliters of culture medium was introduced into the microplate wells. Next, 10 *μ*L of the aqueous extract of *P. biglobosa* (5, 10, 19, 40, 77, 153, 306, 615, 1225, and 2450 *μ*g/mL) was added to all wells, except for the control (culture medium) and negative (glutamate only) control wells. One hour later, 20 *μ*L of L-glutamate (0.1 mg/mL) was added to each well. The cells were placed in a controlled incubator (temperature, 37°C, humidity, and 5% CO_2_) for 24 h. On the second day of the experiment, the supernatant from each well was gently removed and replaced with 100 *μ*L of MTT reagent (1 mg/mL). The preparation was incubated again at 37°C for 1 h. After incubation, 50 *μ*L dimethylsulfoxide was added to the wells to dissolve the formazan crystals resulting from MTT conversion by mitochondrial succinate dehydrogenase. The mixture was homogenized and vortexed for 10 min. Percentage cell viability was determined by measuring optical density with a spectrophotometer at 540 nm. Percentage of cell viability was calculated according to the following formula:

 $$\begin{array}{c} \text{cell viability }\left(\%\right)=100\times \frac{\left(\text{OD of culture treated by L}‐\text{glutamate}+PB-\text{OD of culture treated by L}‐\text{glutamate}\right)}{\text{OD of control culture}-\text{OD of culture treated by L}‐\text{glutamate}} \end{array}$$

where OD stands for optical density and *PB* stands for *Parkia biglobosa*.

### 2.9. Quantitative Phytochemical Analysis of *P. biglobosa* Aqueous Extract

The concentration of total polyphenols, total flavonoids, total tannins, alkaloids, and saponins was determined, respectively, by the methods of J. Leutgeb and S. Leutgeb [17], Kesner [18], Lynch et al. [19], and Zammit et al. [20].

### 2.10. Statistics

Statistical analysis of the values obtained and construction of the graphs were carried out using XLSTAT, GraphPad Prism version 8.01, and Microsoft Office Excel 2016 version 15.0.4420.1017. Results were expressed as mean ± standard error of the mean (SEM) or as percentages. The different values were compared using the analysis of variance (one-way ANOVA) test followed by Tukey's multiple comparison test to highlight the significance between the different groups. Fisher's exact probability was used to compare percentages. From *p* < 0.05, differences were considered significant.

## 3. Results

### 3.1. Effects of *P. biglobosa* Aqueous Extract on Recognition of Objects in the Open Arena

**Figure 2:**
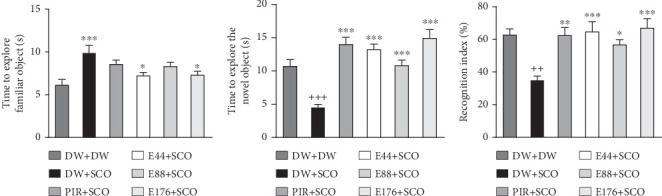
Effects of *Parkia biglobosa* aqueous extract on familiar object exploration time (a), new object exploration time (b), and recognition index (c). Each bar represents the mean ± SEM, *n* = 7. ^++^*p* < 0.01 and ^+++^*p* < 0.001: significant difference compared with the normal control group (DW + DW). ⁣^∗^*p* < 0.05, ⁣^∗∗^*p* < 0.01, and ⁣^∗∗∗^*p* < 0.001: significant difference compared with negative control (DW + SCO). DW + DW: normal control; DW + SCO: negative control; PIR + SCO: positive controls treated with piracetam (200 mg/kg); E44 + SCO, E88 + SCO, and E176 + SCO: test groups treated with 44, 88, and 176 mg/kg extract, respectively.

Figure 2 shows the effects of *P. biglobosa* aqueous extract on familiar object exploration time, new object exploration time, and new object RI in the open arena.

SCO induced an increase (*p* < 0.001) in familiar object exploration time (Figure 2(a)) and a decrease (*p* < 0.001) in new object exploration time in the negative control group compared with the normal control group (Figure 2(b)).

Extract (44 and 176 mg/kg) decreased familiar object investigation time by 15.59% (*p* < 0.05) and 15.11% (*p* < 0.05), respectively, compared with the negative control group (Figure 2(a)). Extract at doses of 44, 88, and 176 mg/kg induced an increase in this exploration of the novel object by 48.87% (*p* < 0.001), 40.81% (*p* < 0.001), and 53.31% (*p* < 0.001) compared with the negative control group (Figure 2(b)).

Piracetam induced a nonsignificant decrease in exploration time for the familiar object (Figure 2(a)) but induced a 61% (*p* < 0.001) increase in investigation time for the novel object compared with the negative control group (Figure 2(b)).

Intraperitoneal administration of SCO leads to a decrease (*p* < 0.01) in the RI in the negative control group compared with the normal control group (Figure 2(c)). Extract at doses of 44 and 176 mg/kg increased the RI by 67.5% (*p* < 0.001) and 73.8% (*p* < 0.001), respectively, compared with the normal control group.

### 3.2. Effects of the Aqueous Extract of *P. biglobosa* on Working Memory in the T-Maze

**Figure 3:**
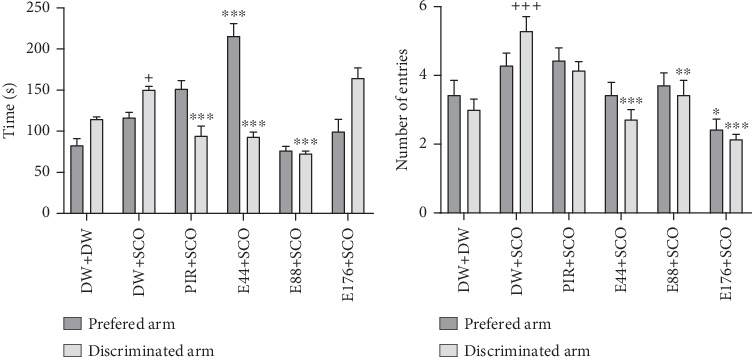
Effects of *Parkia biglobosa* aqueous extract on time spent (a) and number of entries (b) in preferred and discriminated arms of the T-maze. Each bar represents the mean ± SEM, *n* = 7. ^++^*p* < 0.01: significant difference compared with the normal control group (DW + DW). ⁣^∗^*p* < 0.05; ⁣^∗∗^*p* < 0.01; ⁣^∗∗∗^*p* < 0.001: significant difference compared with negative control (DW + SCO). DW + DW: normal control; DW + SCO: negative control; PIR + SCO: positive control treated with piracetam (200 mg/kg); E44 + SCO, E88 + SCO, and E176 + SCO: test groups treated with 44, 88, and 176 mg/kg extract, respectively.

Figure 3 shows the effects of the aqueous extract of *P. biglobosa* on time spent (Figure 3(a)) and the number of entries (Figure 3(b)) in the preferred and discriminated arms of the T-maze.

SCO induced a significant (*p* < 0.05) increase in time spent in the discriminated arm compared with the normal control group. Extract at a dose of 44 mg/kg induced a 29.86% (*p* < 0.001) increase in time spent in the preferred arms (Figure 3(a)) and a 23.53% (*p* < 0.001) decrease in time in the discriminated arm compared with the negative control group. In addition, piracetam decreased the number of entries into the discriminated arm by 22.91%.

SCO induced an increase (*p* < 0.001) in the number of entries into the discriminated arm compared with the normal control group (Figure 3(b)). The extract (44, 88, and 176 mg/kg) induced a decrease of 32.15% (*p* < 0.001), 21.30% (*p* < 0.01), and 42.30% (*p* < 0.001), respectively, in the number of entries into the discriminated arms in the groups receiving the extract at doses of 44 and 176 mg/kg compared with the negative control group.

### 3.3. Effects of the Aqueous Extract of *P. biglobosa* on the Working and Spatial Memories in the Radial Maze

#### 3.3.1. Effect on the Working Memory

**Figure 4:**
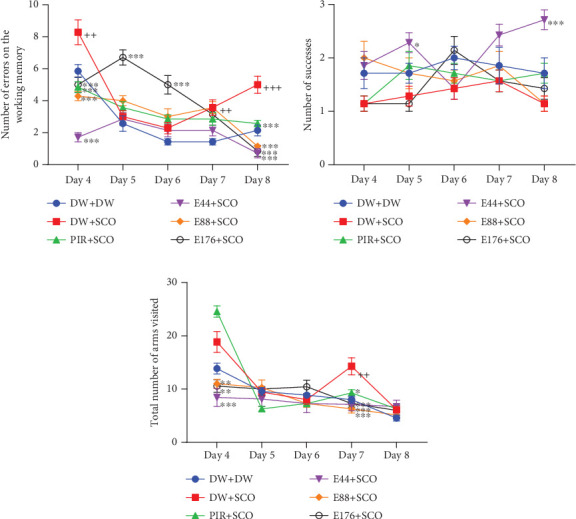
Effects of *Parkia biglobosa* aqueous extract on the number of reference memory errors (a), the number of successes (b), and the total number of arms visited (c) in the radial arm maze. Each bar represents the mean ± SEM, *n* = 7. ^++^*p* < 0.01 and ^+++^*p* < 0.001: significant difference compared with the normal control group (DW + DW). ⁣^∗∗∗^*p* < 0.001: significant difference compared with negative control (DW + SCO). DW + DW: normal control; DW + SCO: negative control; PIR + SCO: positive control treated with piracetam (200 mg/kg); E44 + SCO, E88 + SCO, and E176 + SCO: test groups treated with 44, 88, and 176 mg/kg extract, respectively.

Figure 4 illustrates the effect of *P. biglobosa* aqueous extract on working memory. It shows that intraperitoneal injection of SCO led to an increase in the number of working memory errors (Figure 4(a)) on Days 4 (*p* < 0.01), 7 (*p* < 0.01), and 8 (*p* < 0.001) compared to the normal control group.

The extract decreased the number of working memory errors from Day 4 to Day 8. However, on Day 8, extract (44, 88, and 176 mg/kg) decreased this parameter remarkably, by 85.71% (*p* < 0.001), 77.14% (*p* < 0.001), and 82.85% (*p* < 0.001), respectively, compared with the negative control group.

Piracetam induced a reduction in the number of working memory errors from Day 4 to Day 8. The latter showed a remarkable decrease of 32.08% (*p* < 0.001) on Day 8 compared with the negative control group (Figure 4(a)).

SCO induced a nonsignificant decrease in the number of successes (Figure 4(b)) in the negative control group compared with the normal control group. The 44 mg/kg extract induced an increase in the number of successes from Day 4 to Day 8. Furthermore, extract (44 mg/kg) increased this parameter by 40.73% (*p* < 0.001) on Day 8. However, piracetam induced a nonsignificant increase in the number of successes compared with the negative control group (Figure 4(b)).

Intraperitoneal administration of SCO induced an increase in the total number of arms visited (Figure 4(c)) in the negative control group compared with the positive control group, especially on Day 7 (*p* < 0.01). The aqueous extract of *P. biglobosa* bark at all doses induced a significant decrease in the total number of arms visited in the radial maze arms from Day 4 to Day 8. Indeed, the extract (44, 88, and 176 mg/kg) on Day 7 particularly induced a decrease in this parameter of 50.01% (*p* < 0.001), 56.01% (*p* < 0.001), and 49.01% (*p* < 0.001), respectively, compared with the negative control group. In addition, piracetam induced an overall decrease in the parameter studied, particularly by 35.01% (*p* < 0.05) on Day 7.

#### 3.3.2. Effect on Spatial Memory

**Figure 5:**
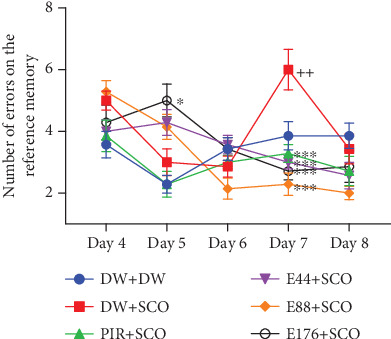
Effects of *Parkia biglobosa* aqueous extract on reference memory in the radial arm maze. Each bar represents the mean ± SEM, *n* = 7. ^+^*p* < 0.05: significant difference compared with the normal control group (DW + DW). ⁣^∗^*p* < 0.05 and ⁣^∗∗∗^*p* < 0.001: significant difference compared with negative control (DW + SCO). DW + DW: normal control; DW + SCO: negative control; PIR + SCO: positive controls treated with piracetam (200 mg/kg); E44 + SCO, E88 + SCO, and E176 + SCO: test groups treated with 44, 88, and 176 mg/kg extract, respectively.

Figure 5 illustrates the effect of *P. biglobosa* extract on spatial memory. SCO induced a significant increase in the number of reference memory errors in the negative control group (*p* < 0.01) compared with the normal control group, particularly on Day 7. The extract induced a decrease in the number of reference memory errors from Day 4 to Day 8. However, the extract (44, 88, and 176 mg/kg) decreased this parameter compared with the negative control group by 50% (*p* < 0.001), 61.9% (*p* < 0.001), and 54.76% (*p* < 0.001), respectively. In addition, piracetam induced a decrease in this parameter, particularly by 29.22% (*p* < 0.001) on Day 7 compared with the negative control group.

### 3.4. Effects of *Parkia biglobosa* Aqueous Extract on the Concentration of ACh

**Figure 6:**
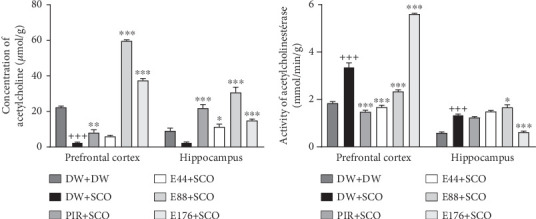
Effects of *Parkia biglobosa* aqueous extract on acetylcholine concentration (a) and acetylcholinesterase activity (b) in the prefrontal cortex and hippocampus. Each bar represents the mean ± SEM, *n* = 7. ^+++^*p* < 0.001: significant difference compared with the normal control group (DW + DW). ⁣^∗^*p* < 0.05, ⁣^∗∗^*p* < 0.01, and ⁣^∗∗∗^*p* < 0.001: significant difference compared with negative control (DW + SCO). DW + DW: normal control; DW + SCO: negative control; PIR + SCO: positive controls treated with piracetam (200 mg/kg); E44 + SCO, E88 + SCO, and E176 + SCO: test groups treated with 44, 88, and 176 mg/kg extract, respectively.

Figure 6 shows the effects of *P. biglobosa* extract on ACh concentration (Figure 6(a)) and AChE activity (Figure 6(b)) in the cerebral cortex and hippocampus.

Compared with the normal control group, SCO led to a significant decrease in ACh (Figure 6(a)) in the cortex (*p* < 0.001), but not significantly in the hippocampus. The 88 and 176 mg/kg extracts showed an increase compared with the negative control group of 95.79% (*p* < 0.001) and 93.28% (*p* < 0.001), respectively, in the prefrontal cortex. In the hippocampus, extract at doses of 44, 88, and 176 mg/kg showed an increase in ACh compared with the negative control group of 77.94% (*p* < 0.05), 91.89% (*p* < 0.001), and 83.27% (*p* < 0.001), respectively. Furthermore, piracetam (Figure 6(a)) induced an increase in ACh in the positive control group compared with the negative control group in the prefrontal cortex by 69.02% (*p* < 0.01) and in the hippocampus by 88.64% (*p* < 0.001).

Compared with the normal control group, SCO induced a significant increase in AChE activity (Figure 6(b)) in the prefrontal cortex (*p* < 0.001) and hippocampus (*p* < 0.001). The extract, particularly at the 44 mg/kg dose, induced a decrease in this parameter in comparison with the 44 mg/kg dose.

### 3.5. Effects of *P. biglobosa* Aqueous Extract on the Concentration of GABA

**Figure 7:**
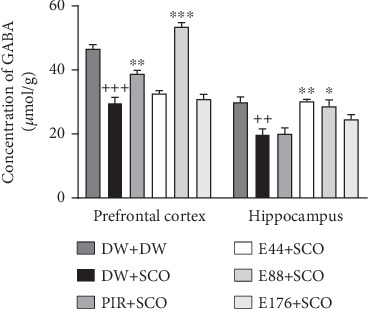
Effects of *Parkia biglobosa* aqueous extract on gamma amino butyric acid concentration in cortex and hippocampus. Each bar represents the mean ± SEM, *n* = 7. ^++^*p* < 0.01 and ^+++^*p* < 0.001: significant difference compared to the normal control group (DW + DW). ⁣^∗^*p* < 0.05, ⁣^∗∗^*p* < 0.01, and ⁣^∗∗∗^*p* < 0.001: significant difference compared with negative control (DW + SCO). DW + DW: normal control; DW + SCO: negative control; PIR + SCO: positive controls treated with piracetam (200 mg/kg); E44 + SCO, E88 + SCO, and E176 + SCO: test groups treated with 44, 88, and 176 mg/kg extract, respectively.

Figure 7 shows the effects of *P. biglobosa* on GABA concentration in the prefrontal cortex and hippocampus. SCO induced a 36.12% (*p* < 0.001) decrease in GABA in the prefrontal cortex and a 33.43% (*p* < 0.005) decrease in the hippocampus compared with the normal control group. The aqueous extract of *P. biglobosa* increased GABA concentration in the cortex by 44.35% (*p* < 0.01) compared with a negative control group at a dose of 88 mg/kg. In the hippocampus, the extract (44 mg/kg) induced an increase of 34.20% (*p* < 0.05) compared with the negative control group. Piracetam induced a significant increase in GABA in the prefrontal cortex of 23.27% (*p* < 0.01), but a nonsignificant increase in the hippocampus compared with the negative control group.

### 3.6. Effects of *P. biglobosa* Aqueous Extract on Oxidative Stress Parameters

**Figure 8:**
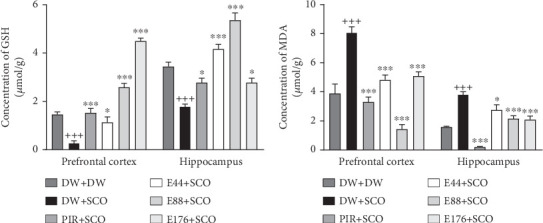
Effects of the aqueous extract of *Parkia biglobosa* on the concentration of reduced glutathione (a) and malondialdehyde (b) in the prefrontal cortex and hippocampus. Each bar represents the mean ± SEM, *n* = 7. ^++^*p* < 0.01 and ^+++^*p* < 0.001: significant difference compared to the normal control group (DW + DW). ⁣^∗^*p* < 0.05, ⁣^∗∗^*p* < 0.01, and ⁣^∗∗∗^*p* < 0.001: significant difference compared with negative control (DW + SCO). DW + DW: normal control; DW + SCO: negative control; PIR + SCO: positive controls treated with piracetam (200 mg/kg); E44 + SCO, E88 + SCO, and E176 + SCO: test groups treated with 44, 88, and 176 mg/kg extract, respectively.

Figure 8 shows the effects of *P. biglobosa* on GSH and MDA concentration in the prefrontal cortex and hippocampus of rats.

SCO administration resulted in a significant decrease (*p* < 0.001) in GSH content (Figure 8(a)) in the prefrontal cortex and hippocampus compared with the normal control. The extract (44, 88, and 176 mg/kg) increased GSH concentration in the cortex by 76.01% (*p* < 0.05), 89.49% (*p* < 0.001), and 93.97% (*p* < 0.001), respectively, compared with the negative control group. In the hippocampus, the extract (44, 88, and 176 mg/kg) induced an increase of 57.07% (*p* < 0.001), 66.60% (*p* < 0.001), and 35.61% (*p* < 0.05), respectively. In addition, piracetam induced an 82.17% (*p* < 0.001) increase in GSH in the prefrontal cortex and a 35.61% (*p* < 0.001) increase in the hip.

SCO induced a significant increase in MDA (Figure 8(b)) in the negative control group compared with the normal control group of 51.91% (*p* < 0.001) in the prefrontal cortex and 58.71% (*p* < 0.001) in the hippocampus. In the prefrontal cortex, the extract (44, 88, and 176 mg/kg) induced a reduction of 40.36% (*p* < 0.001), 82.27% (*p* < 0.001), and 37.15% (*p* < 0.001), respectively. In the hippocampus, the extract (44, 88, and 176 mg/kg) induced a significant decrease of 28.15% (*p* < 0.05), 43.79% (*p* < 0.001), and 45.38% (*p* < 0.001), respectively. Piracetam induced a 59.15% (*p* < 0.001) decrease in the prefrontal cortex and a 94.65% (*p* < 0.001) decrease in the hippocampus (Figure 8(b)).

### 3.7. Effects of *P. biglobosa* Aqueous Extract on Some Proinflammatory Cytokines

**Figure 9:**
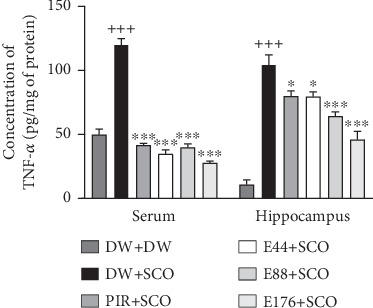
Effects of *Parkia biglobosa* aqueous extract on TNF-*α* concentration in serum and the hippocampus. Each bar represents the mean ± SEM, *n* = 7. ^+++^*p* < 0.001: significant difference compared to the normal control group (DW + DW). ⁣^∗^*p* < 0.05 and ⁣^∗∗∗^*p* < 0.001: significant difference compared with negative control (DW + SCO). DW + DW: normal control; DW + SCO: negative control; PIR + SCO: positive control treated with piracetam (200 mg/kg); E44 + SCO, E88 + SCO, and E176 + SCO: test groups treated with 44, 88, and 176 mg/kg extract, respectively.

Figure 9 shows the effects of *P. biglobosa* extract on TNF-*α* concentration in serum and the hippocampus. SCO induced a significant increase in serum TNF-*α* (*p* < 0.001) and hippocampal TNF-*α* (*p* < 0.001) compared to the normal control group. Extract at doses of 44, 88, and 176 mg/kg showed a decrease in TNF-*α* compared with the negative control group of 71.04% (*p* < 0.05), 66.89% (*p* < 0.001), and 76.92% (*p* < 0.001) in serum. In the hippocampus, the extract at doses of 44, 88, and 176 mg/kg induced a decrease in TNF-*α* compared with the negative control group of 23.89% (*p* < 0.05), 38.54% (*p* < 0.001), and 56.08% (*p* < 0.001), respectively. We also observed a decrease in TNF-*α* in the positive control group compared with the negative control group in serum of 65.39% (*p* < 0.001) and 23.65% (*p* < 0.05) in the hippocampus.

### 3.8. Effects of *P. biglobosa* Aqueous Extract on Neuronal Microarchitecture

**Figure 10:**
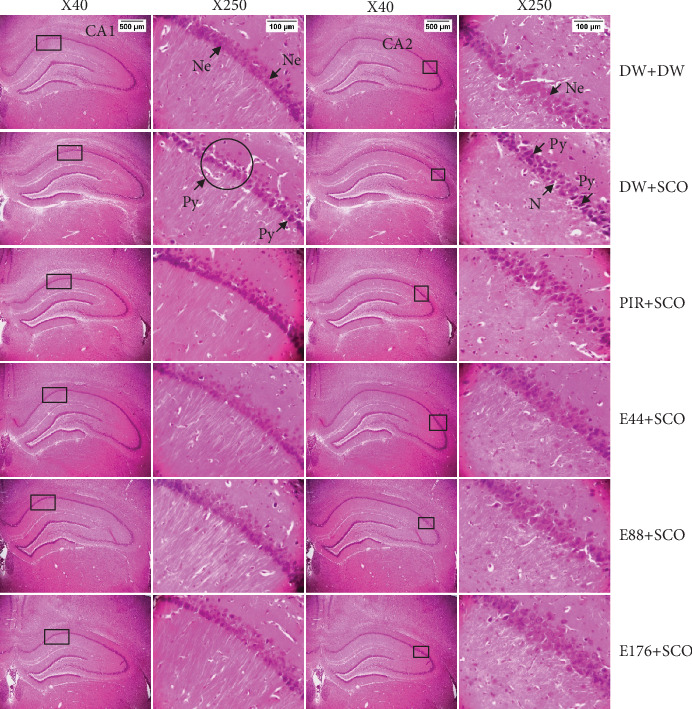
Microphotographs of CA1 and CA2 showing the effects of *Parkia biglobosa* aqueous extract on neuronal microarchitecture (hematoxylin–eosin staining). DW + DW = normal control; DW + SCO = negative control; PIR + SCO = positive control treated with piracetam (200 mg/kg); E44 + SCO, E88 + SCO, and E176 + SCO = aqueous extract of *P. biglobosa* at doses of 44, 88, and 176 mg/kg, respectively; Py = pycnosis; Nc = cytolyzed neuron. × 250 columns correspond to boxed areas representing hippocampal CA1 and CA2, respectively. This magnification was used to count neurons.

**Figure 11:**
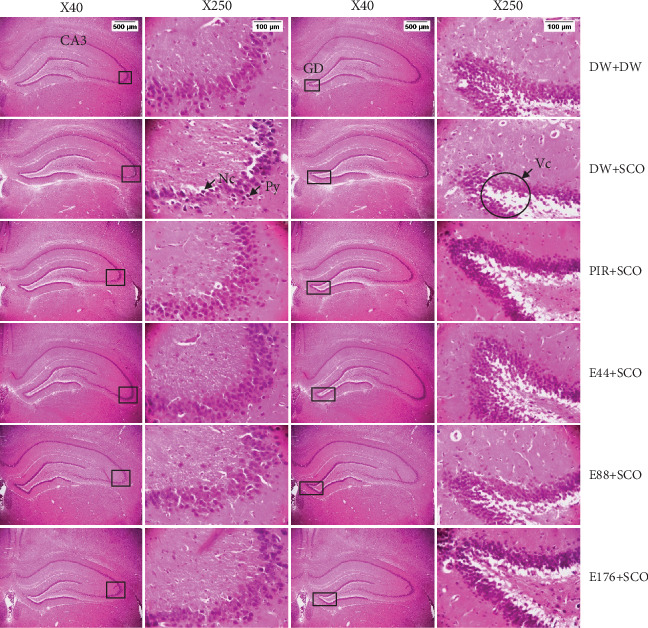
Microphotographs of CA3 and dentate gyrus showing the effects of *Parkia biglobosa* aqueous extract on neuronal microarchitecture (hematoxylin–eosin staining). DW + DW = normal control; DW + SCO = negative control; PIR + SCO = positive control treated with piracetam (200 mg/kg); E44 + SCO, E88 + SCO, and E176 + SCO = aqueous extract of *P. biglobosa* at doses of 44, 88, and 176 mg/kg, respectively; Py = pycnosis; Nc = cytolyzed neuron, Vc = vacuole of cell. The × 250 columns correspond to the framed areas representing CA3 and the dentate gyrus of the hippocampus, respectively. At this magnification, neurons can be counted.

**Figure 12:**
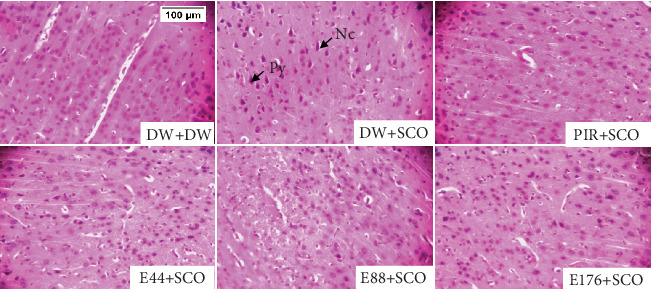
Microphotographs of the prefrontal cortex showing the effects of *Parkia biglobosa* aqueous extract on neuronal microarchitecture (hematoxylin–eosin staining). DW + DW = normal control; DW + SCO = negative control; PIR + SCO = positive control treated with piracetam (200 mg/kg); E44 + SCO, and E88 + SCO, E176 + SCO = aqueous extract of *P. biglobosa* at doses of 44, 88, and 176 mg/kg, respectively; Py = pycnosis; Nc = cytolyzed neuron. × 250 columns represent the prefrontal cortex. This magnification enabled neurons to be counted.

Figures 10, 11, and 12 show the effects of *P. biglobosa* aqueous bark extract on neuronal microarchitecture.

Histological analysis showed normal hippocampal and prefrontal cortex structure in normal control animals. In animals in the negative control group, compared with the normal control group, a loss of neuronal tissue integrity was observed, marked by the presence of neurons with pycnosed (hyperchromatic) nuclei, neuronal loss, and cytolysis. The extract (44, 88, and 176 mg/kg) and piracetam groups showed a microstructure of the hippocampus and prefrontal cortex close to that of the normal control group.

### 3.9. Effects of *P. biglobosa* Aqueous Extract on Neuronal Density in the Hippocampus and Prefrontal Cortex

**Figure 13:**
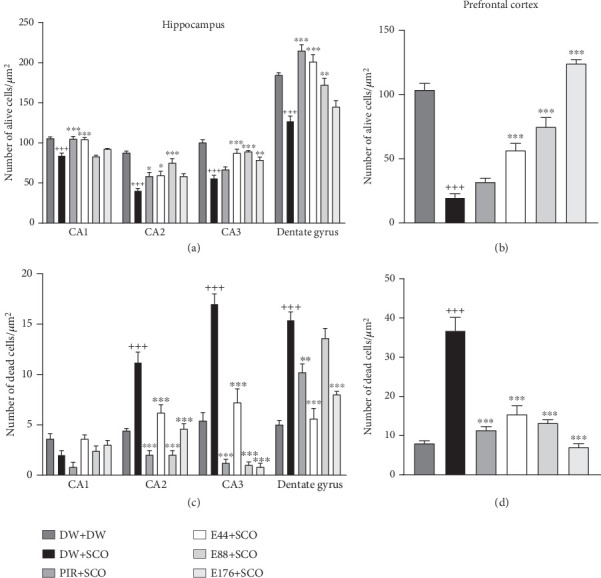
Effects of *Parkia biglobosa* aqueous extract on alive cells (a, b) and dead cells (c, d) in the hippocampus and prefrontal cortex. Each bar represents the mean ± SEM, *n* = 5. ^+++^*p* < 0.001: significant difference compared with the normal control group (DW + DW). ⁣^∗^*p* < 0.05 and ⁣^∗∗∗^*p* < 0.001: significant difference compared with negative control (DW + SCO). DW + DW: normal control; DW + SCO: negative control; PIR + SCO: positive control treated with piracetam (200 mg/kg); E44 + SCO, E88 + SCO, and E176 + SCO: test groups treated with 44, 88, and 176 mg/kg extract, respectively.

Figure 13 illustrates the effects of *P. biglobosa* aqueous extract on the number of alive cells (Figures 13(a) and 13(b)) and dead cells (Figures 13(c) and 13(d)) in the hippocampus and prefrontal cortex.

SCO induced a decrease in the number of live cells in area CA1 by 11.27% (*p* < 0.001), CA2 by 36.87% (*p* < 0.001), CA3 by 28.38% (*p* < 0.001), dentate gyrus by 18.35% (*p* < 0.001), and prefrontal cortex by 67.90% (*p* < 0.001) compared with the normal control group (Figures 13(a) and 13(b)). The 44 mg/kg extract induced live cell protection in area CA1 by 10.61% (*p* < 0.001), CA2 by 19.03% (*p* < 0.05), CA3 by 21.78% (*p* < 0.001), and prefrontal cortex by 48.03% (*p* < 0.001) compared with the normal control group. Piracetam (Figures 13(a) and 13(b)) protected nerve cells by 10.89% (*p* < 0.001) in CA1, 18.21% (*p* < 0.05) in CA2, and 25.61% (*p* < 0.001) in the dentate gyrus of the hippocampus, respectively, compared with the negative control group.

SCO induced an increase in the number of dead cells in area CA2 by 43.58% (*p* < 0.001), CA3 by 51.78% (p<0.001), dentate gyrus by 50.98% (*p* < 0.001), and prefrontal cortex by 64.28% (*p* < 0.001) (Figures 13(c) and 13(d)). The extract (44 mg/kg) remarkably limited neuronal loss in these zones.

### 3.10. In Vitro Effects of *P. biglobosa* Aqueous Extract on Cell Viability

**Figure 14:**
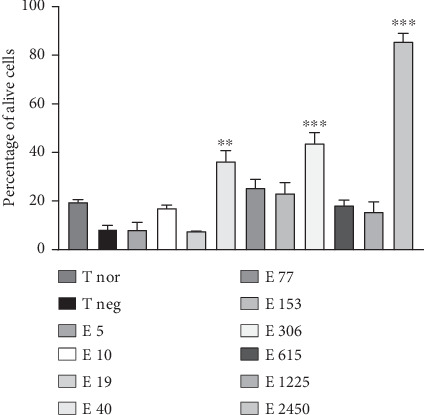
Effects of *Parkia biglobosa* aqueous extract on cortical nerve cell viability. Each bar represents the mean ± SEM, *n* = 4. ⁣^∗∗∗^*p* < 0.001: significant difference compared with the negative control group (T neg). T nor: normal control; T neg: negative control; E 5; E 10, E 19, E 40, E 77, E 153, E 306, E 615, E 1225, and E 2450 test groups treated with 5, 10, 19, 40, 77, 153, 306, 615, 1225, and 2450 *μ*g/mL extract, respectively.

Figure 14 illustrates the effect of the extract on the percentage of viable nerve cells. L-Glutamate induced a nonsignificant decrease in the percentage of viable cells compared with the normal control group. The extract (40, 306, and 2450 *μ*g/mL) induced a 62.46% (*p* < 0.01), 67.80% (*p* < 0.001), and 82.12% (*p* < 0.001) increase in the percentage of viable cells, respectively, compared with the negative control group (Figure 14).

### 3.11. Quantitative Phytochemistry of the Aqueous Extract of *P. biglobosa*

The quantitative phytochemical screening of *P. biglobosa* extract is presented in Table 2. The extract was found to be high in alkaloids, flavonoids, and polyphenols. However, the extract was low in saponins and tannins.

## 4. Discussion

Our study is aimed at evaluating the antiamnesic and neurotrophic effects of the aqueous extract of *P. biglobosa* using *in vivo* and *in vitro* models of excitotoxicity. Synaptic deficit is one of the primary features of memory impairment and is closely linked to reduced cognitive function and memory [21]. Numerous studies have shown that SCO is a muscarinic receptor antagonist and is capable of producing deficits in learning, acquisition, and consolidation [21]. Similarly, high oxidative stress triggers a vicious circle of synaptic dysfunction, memory deficit, neuroinflammation, and apoptosis. Several studies thus support the use of SCO as a good model for the induction of memory disorders [22].

The new object recognition test was conducted to assess short-term memory. In the in vivo study, SCO led to a decrease in the new object RI in the negative control group. This reflects an impairment of short-term memory. These results are similar to studies by Huang et al. [23] who showed that SCO impairs ACh transmission in the prefrontal cortex by blocking M1 muscarinic receptors. Nevertheless, the aqueous extract showed an increase in the index of recognition of the new object, suggesting an antiamnesic effect against short-term memory disorders. This increase would be due to the presence of flavonoids quantified in *P. biglobosa* extract. Indeed, these secondary metabolites have an inhibitory action on AChE [24].

The T-maze test was used in the present study to assess short-term memory in support of the test performed above. SCO induced an increase in time spent and number of entries in the discriminated arm and a decrease in the number of entries in the preferred arm in the negative control group. These results support the impact of SCO on short-term memory [25]. Cholinergic transmission plays an important role in memory and cognitive function [26]. In cholinergic dysfunction, AChE is responsible for the degradation of ACh into acetate and choline and reduces neurotransmitters in the brain. Consequently, abnormal overexpression of AChE leads to impaired memory and cognition via cholinergic dysfunction memory and cognition via cholinergic dysfunction [26]. The extract induced an increase in the time and number of entries by rats into the preferred arm compared with the discriminated arm. The presence of these antiamnesic effects is thought to be due to the presence of flavonoids and polyphenols, quantified in *P. biglobosa* extract, which has an inhibitory activity on AChE and antioxidant power by reducing the formation of free radicals [24, 27].

The radial arm maze test is used to assess short- and long-term memory. In the present study, SCO induced an increase in the number of errors in working memory and reference memory. This indicates impairment of both short- and long-term memory. Muscarinic M1 receptors are highly expressed in the hippocampus and their inhibition or ablation disrupts spatial memory encoding [28]. In the present study, the aqueous extract of *P. biglobosa* led to a reduction in the number of errors in working memory and reference memory. These results suggest an antiamnesic effect of *P. biglobosa* on short- and long-term memory. These effects are thought to be due to the presence of flavonoids and polyphenols quantified in the extract for their inhibitory actions on AChE [27].

According to several previous publications, intraperitoneal injection of SCO led to an attenuation of cholinergic, high oxidative stress, and oxidative and inflammatory [29]. In the present study, SCO induced in the brain a decrease in ACh, an increase in AChE activity, and a decrease in GABA. The work of Gallardo and Holtzman [30] has consistently established the cotransmission of ACh and GABA in the hippocampus via synapses. Synapses do not corelease but cotransmit GABA and ACh via different vesicles, whose release is triggered by distinct calcium channels [30]. In this study, the extract induced an increase in ACh, a decrease in AChE, and a decrease in GABA. The increase in ACh is thought to be due to the presence of flavonoids with AChE inhibitory activity [24].

SCO is a muscarinic cholinergic antagonist known to damage learning and memory by disrupting cholinergic transmission [31]. In this study, the amount of reduced GSH and MDA in the prefrontal cortex and hippocampus was assessed. SCO induced a decrease in GSH and an increase in MDA. GSH is an essential tripeptide, an antioxidant present in all animal cells. It protects cells from damage caused by oxygen, superoxide radicals, and the hydroxyl radical [50]. MDA is a product of lipid peroxidation. Free radicals capture electrons from lipid molecules inside the cell membrane, leading to peroxidation of lipid molecules inside the cell membrane [50]. MDA indicates the state of oxidative damage to membranes under conditions of oxidative stress [50]. In the course of this work, it was observed that the aqueous extract of *P. biglobosa* induced a significant decrease in MDA and an increase in GSH. These results are thought to be due to the presence of polyphenols and alkaloids with antioxidant activity [32].

According to the work of Hussain et al. [33], in addition to cognitive function, ACh is a key component of the cholinergic anti-inflammatory system through the regulation of TNF-*α*. It also triggers and induces nerve cell and synapse death, contributing to neurodegeneration [33]. SCO treatment increased TNF-*α* levels in the negative control group compared with the normal control group [33]. Aqueous extract of *P. biglobosa* decreased TNF-*α* levels in both the hippocampus and serum. According to the work of Muhammad et al. [34], the decrease in TNF-*α* could reflect a cerebral anti-inflammatory effect of the plant through the presence of flavonoids and tannins quantified in the extract [34, 35].

The memory impairment observed in the present work would be the result of histological alterations in the hippocampus and prefrontal cortex [36]. In the present study, intraperitoneal administration of SCO resulted in neuronal loss in the CA1, CA2, CA3, dentate gyrus, and prefrontal cortex. These results are similar to those of Tripathi, Paliwal, and Krishnamurthy [37], who showed disorganization of these brain regions in memory disorders. *P. biglobosa* extract prevented neuronal loss. This result suggests antioxidant, antiamnesic, and neuroprotective activity [7, 37].

Neurogenesis arises from neuronal stem cells located in the dentate gyrus of the hippocampus [38]. In our study, the choice of embryos to assess neurogenesis was aimed at obtaining a higher probability of obtaining stem cells [39]. According to the work of Quansah et al. [40], the purpose of the cell viability test is to assess the cytotoxicity or capacity of the extract to produce new cells. In this study, L-glutamate induced a decrease in the number of viable cells. Indeed, according to the work of Odounharo et al. [41], L-glutamate induces degeneration and growth arrest of nerve cells through excitotoxicity. The extract induced a considerable increase in viable nerve cells. These results suggest the plant's ability to induce neurogenesis through neurotrophic effects, and this could be due to the presence of flavonoids, polyphenols, and alkaloids quantified in *P. biglobosa* extract [42].

## 5. Conclusions

The present study is aimed at evaluating the antiamnesic and neurotrophic effects of the aqueous extract of *P. biglobosa* on a SCO-induced model of memory disorders in Wistar rats. SCO induced memory impairment, oxidative stress, neuroinflammation, and neuronal loss in the hippocampus and prefrontal cortex. Phytochemical screening of the extract revealed the presence of secondary metabolites such as alkaloids, flavonoids, polyphenols, saponins, and tannins.

Treatment with the aqueous extract of *P. biglobosa* (44 mg/kg) reduced the number of errors in working memory and reference memory. These results suggest that the extract corrects memory disorders. The extract significantly increased the concentration of some neurotransmitters (ACh and GABA), resulting in improved memorization. The extract significantly increased the concentration of reduced glutathione and remarkably reduced MDA levels. These results suggest that the extract has antioxidant activity. The aqueous extract of *P. biglobosa* significantly decreased TNF-*α* concentration, revealing anti-inflammatory activity. Histological sections of the hippocampus and prefrontal cortex showed that *P. biglobosa* aqueous extract was able to limit neuronal loss, indicating a neuroprotective effect. Furthermore, the cell viability test showed that the extract (2450 *μ*g/mL) is capable of inducing the production of new neurons, reflecting a neurotrophic effect.

Taken together, these observations suggest that *P. biglobosa* has antiamnesic and neurotrophic properties mediated by its neuromodulatory, antioxidant, and anti-inflammatory activities. These results could justify the use of *P. biglobosa* extract in traditional medicine for the prevention of memory disorders and other neurological diseases.

## Figures and Tables

**Table 1:** Distribution and treatment of rats.

**Groups**	**Nb rats**	**Treatment phase (1st administration)**	**Latency**	**Induction phase (2nd administration)**	**Coadministration (**1**st** + 2**nd)**	**Abbreviation**
Normal control group	7	Distilled water (10 mL/kg, po)	45 min	Distilled water (10 mL/kg, ip)	Once daily for 15 days	DW + DW

Negative control group	7	Distilled water (10 mL/kg, po)	45 min	Scopolamine (0.5 mg/kg, ip)	Once daily for 15 days	DW + SCO

Test groups	7	*P. biglobosa* (44 mg/kg)	45 min	Scopolamine (0.5 mg/kg, ip)	Once daily for 15 days	E44 + SCO
	7	*P. biglobosa* (88 mg/kg)	45 min	Scopolamine (0.5 mg/kg, ip)	Once daily for 15 days	E88 + SCO
	7	*P. biglobosa* (176 mg/kg)	45 min	Scopolamine (0.5 mg/kg, ip)	Once daily for 15 days	E176 + SCO

Positive control group	7	Piracetam (200 mg/kg, po)	45 min	Scopolamine (0.5 mg/kg, ip)	Once daily for 15 days	PIR + SCO

Abbreviations: 1st = first administration, 2nd = second administration, DW = distilled water, E = extract, ip = intraperitoneally, Nb = number, *P. biglobosa* = *Parkia biglobosa*, PIR = piracetam, po = per os, SCO = scopolamine.

**Table 2:** Quantitative analysis of secondary metabolites in the aqueous extract of *Parkia biglobosa.*

**Metabolites**	**Polyphenols (*μ*g GEA/mg DM)**	**Flavonoids (*μ*g QE/mg DM)**	**Tannins (*μ*g EAG/mg DM)**	**Alkaloids (*μ*g QiE/mg DM)**	**Saponins (*μ*g SaE/mg DM)**
	182.72 ± 1.97	183.89 ± 1.92	0.09 ± 0.01	626.20 ± 16.06	91.91 ± 12.47

*Note:* Values are expressed as mean ± SD.

Abbreviations: CS: concentration of solution; DM: dry matter; GEA: gallic acid equivalent; mg: milligram; QE: quercetin equivalent; QiE: quinine equivalent; SaE: saponin equivalent.

## Data Availability

The datasets used and/or analyzed during the current study are available from the corresponding author on simple request.
